# Remote *in vivo* stress assessment of aquatic animals with microencapsulated biomarkers for environmental monitoring

**DOI:** 10.1038/srep36427

**Published:** 2016-11-03

**Authors:** Anton Gurkov, Ekaterina Shchapova, Daria Bedulina, Boris Baduev, Ekaterina Borvinskaya, Igor Meglinski, Maxim Timofeyev

**Affiliations:** 1 Irkutsk State University, Institute of Biology, Irkutsk, 664003, Russia; 2 Karelian Research Centre of Russian Academy of Sciences, Institute of Biology, Petrozavodsk, 185035, Russia; 3 University of Oulu, Optoelectronics and Measurement Techniques Laboratory, Oulu, 90570, Finland

## Abstract

Remote *in vivo* scanning of physiological parameters is a major trend in the development of new tools for the fields of medicine and animal physiology. For this purpose, a variety of implantable optical micro- and nanosensors have been designed for potential medical applications. At the same time, the important area of environmental sciences has been neglected in the development of techniques for remote physiological measurements. In the field of environmental monitoring and related research, there is a constant demand for new effective and quick techniques for the stress assessment of aquatic animals, and the development of proper methods for remote physiological measurements *in vivo* may significantly increase the precision and throughput of analyses in this field. In the present study, we apply pH-sensitive microencapsulated biomarkers to remotely monitor the pH of haemolymph *in vivo* in endemic amphipods from Lake Baikal, and we compare the suitability of this technique for stress assessment with that of common biochemical methods. For the first time, we demonstrate the possibility of remotely detecting a change in a physiological parameter in an aquatic organism under ecologically relevant stressful conditions and show the applicability of techniques using microencapsulated biomarkers for remote physiological measurements in environmental monitoring.

Remote scanning of various physiological parameters of a living organism is a “dream technology” for medicine and biology. There are great examples of non-invasive techniques to measure specific parameters of an organism, such as the concentration of glucose and alcohol in the blood, blood oxygenation and pulse pressure^1^,^2^,^3^,^4^, based on the optical characteristics of the skin. However, such non-invasive examples still cannot cover the majority of the current needs of the life sciences. Importantly, these techniques are also easily applicable to only humans or mammals and cannot be readily transferred to other animals, for example, invertebrates, which are critically important for the majority of ecosystems and especially for aquatic ecosystems. In this situation, one of the most promising solutions is to implant tiny optical sensors (in other words, artificial biomarkers) into the organism and use them to remotely monitor the parameters of interest in real time and more or less uniformly for different classes of animals.

Fluorescent dyes, whose spectra (i.e., colours) of fluorescence are sensitive to the various chemical and physical characteristics of water solutions^5^, are some of the best candidates to serve as injectable sensors. Although single dyes are usually applicable in subtoxic concentrations^6^,^7^, the effects of many different mixtures of dyes on the organism are laborious to identify and avoid. Because of this, it would be valuable to immobilise fluorescent molecular probes in polymeric microcapsules or microbeads, trapping the dye inside but remaining permeable to metabolites and ions of interest, which keeps the whole sensor sensitive to changes in the organism. Such a shell does not allow the dye to diffuse into the tissues, making the fluorescent signal stronger, and localisable, and eliminating the question of the possible direct influence of fluorescent dye mixtures on the organism.

Despite the fact that areas such as environmental monitoring and ecotoxicology have particular importance for humankind due to the increasing pressures of global climate change and anthropogenic pollution^8^,^9^,^10^, the applicability of remote optical techniques for physiological measurements in these areas has not yet been shown. Environmental monitoring and the related fields of biology have a significant connection to stress assessment, which is based on identification of changes in certain physiological parameters related to physiological stress and the activation of stress response mechanisms. Developing optical techniques for remote stress assessment *in vivo* and in real time would significantly increase the precision and throughput of analyses in these fields.

During the last years, there has been increasing concern about the ecosystem of Lake Baikal and the proper ecological monitoring of this lake^11^,^12^,^13^. Baikal is the oldest and deepest lake in the world^14^, containing 20% of all available surface fresh water on the planet^15^, and is inhabited by extremely diverse endemic fauna^12^,^14^. Because of its uniqueness, Lake Baikal was designated a World Heritage Site by UNESCO. Along with water warming due to the global climate change^12^, an emerging problem of the lake is the massive proliferation of algae and macrophytes in coastal areas, which is caused by local eutrophication and has caused the death of endemic invertebrates of the benthic zone^13^,^16^, where the most animal diversity is found. Therefore, there is an urgent need to develop novel effective techniques for the environmental monitoring of the World’s “Sacred Sea”, which can be later transferred to other aquatic ecosystems.

Both eutrophication and global climate change are connected to alterations in the composition of dissolved gases in water. In particular, these phenomena are significantly related to decreased O_2_ levels (lead to hypoxia) and increased CO_2_ concentration (lead to hypercapnia)^17^,^18^. Hypercapnia and hypoxia in combination with hypercapnia are able to cause the acidification of the internal media of aquatic animals^19^,^20^,^21^,^22^, which can accompany significant disturbances to their metabolism. Thus, the shift in the internal pH in aquatic animals can be used as a stress marker for the conditions of elevated CO_2_ level and decreased O_2_ level.

One of the most important components of the Lake Baikal fauna is amphipods (Amphipoda, Crustacea). They include more than 350 endemic species and subspecies^23^ (the most diverse taxonomical group of the lake and approximately 15% of the biodiversity in the lake) and comprise a significant part of the benthic biomass^24^. The amphipod *Eulimnogammarus verrucosus* (Gerstfeldt, 1858) is one of the most abundant and widespread species in the littoral zone of the lake. This species has been extensively examined in a number of ecophysiological studies^25^,^26^ and is considered a promising general test organism for the environmental monitoring of Lake Baikal littoral zone^27^.

In this study, we apply the technology of microencapsulated biomarkers^28^ (MBMs) to remotely monitor *in vivo* the pH of the haemolymph of the endemic amphipod *E. verrucosus*, and we demonstrate for the first time the remote measurement of an alteration of a physiological parameter induced by stressful conditions. These data were also compared to the results of common biochemical analyses for stress assessment.

## Results

### Signal acquisition from the MBMs injected into amphipods

**Figure 1:**
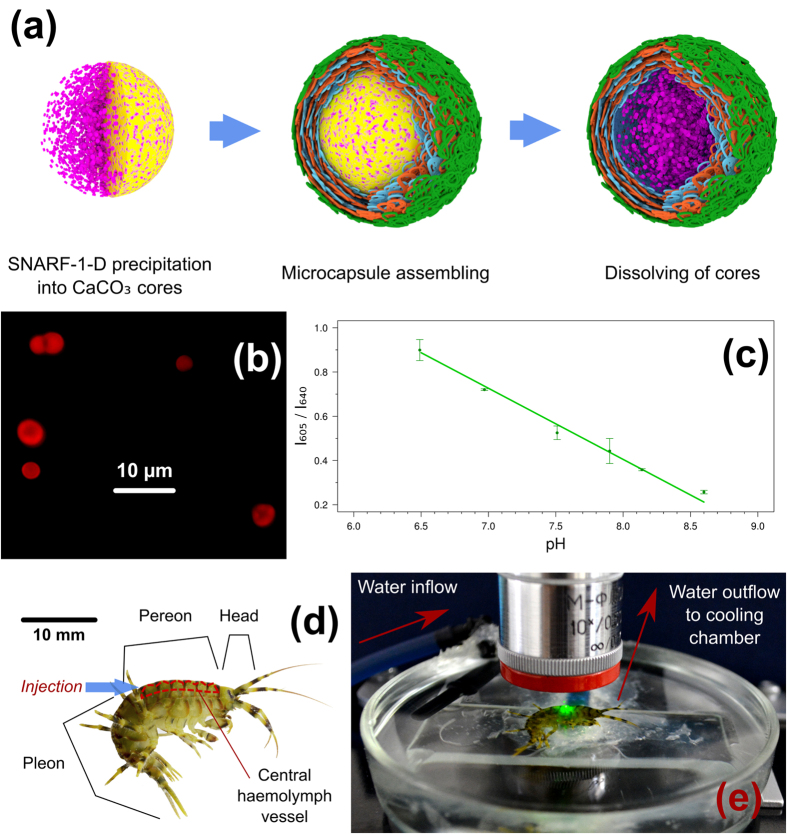
(**a**) General scheme of the preparation of MBMs based on the pH-sensitive fluorescent dye SNARF-1 (here, only 3 layers of positively charged polymer, 3 layers of negatively charged polymer and a final coating of biocompatible polymer are depicted). (**b**) Image of the prepared MBMs recorded using an epifluorescent microscope. (**c**) Calibration curve of the pH-sensitive MBMs at different pHs (mean ± s.d. is depicted). (**d**) *E. verrucosus*, with the main body segments, central haemolymph vessel and point of injection of MBMs indicated. (**e**) System used for amphipod immobilisation under the epifluorescent microscope.

The MBMs used in this work (Fig. 1a,b) are based on the fluorescent dye SNARF-1, which has the best sensitivity to solution pH in the pH range of 6–9^5^. Encapsulated SNARF-1 was used in form of a dextran conjugate (SNARF-1-D) to prevent the diffusion of the dye from the microcapsules, which are permeable to molecules of low molecular weight. SNARF-1 has fluorescence spectra with different ranges of wavelengths depending on its protonation state. The amounts of protonated and deprotonated dye change with pH, and the ratio between the fluorescence intensity at the two wavelengths corresponding to the “acidic” and “basic” ranges is commonly used for pH measurements^5^.

The calibration curve for the prepared MBMs is shown in Fig. 1c. The ratio of the fluorescence intensities at 605 nm and 640 nm was used for calibration, and the MBMs were sensitive to a pH range of at least 6.5–8.6.

**Figure 2:**
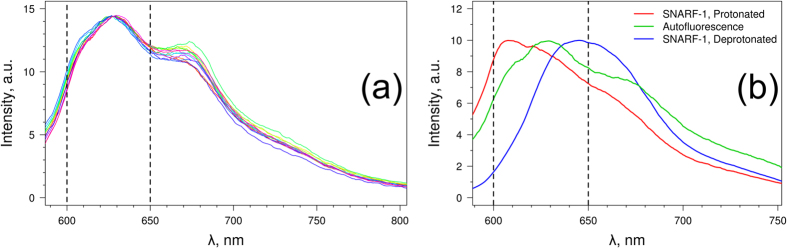
(**a**) Variation in the autofluorescence spectrum of the dorsal part of *E. verrucosus* among different individuals. (**b**) Spectra of protonated SNARF-1 (microencapsulated, at pH 2), deprotonated SNARF-1 (microencapsulated, at pH 8.9) and autofluorescence of *E. verrucosus* used as standards for spectral decomposition with multiple linear regression. The dashed lines show the range used for spectral decomposition.

A common challenge in signal acquisition from a fluorophore of interest inside most organisms is the autofluorescence of the organism’s tissues, which can interfere with the informative fluorescence of the sensor. *In vivo* measurements of pH in amphipods are especially meaningful in the central haemolymph vessel located in the dorsal part of the pereon (Fig. 1d), as any changes in this part of circulatory system are the most representative of the physiological processes in the whole organism. Analysis of *E. verrucosus* autofluorescence in this part of the body showed significant autofluorescence with spectral peaks at 627 nm and 677 nm (Fig. 2a), which are within the functional spectral range of SNARF-1. These autofluorescence spectrum peaks have an unstable ratio with each other over different individuals and parts of the same animal and seem to be produced by different fluorophores. The first autofluorescence peak at 627 nm is probably associated with lipofuscin, an age-related pigment found in crustaceans and many other animals^29^,^30^.

Fortunately, the spectrum of *E. verrucosus* autofluorescence in range of 600–650 nm was stable in the animals analysed (Fig. 2a). Therefore, when the MBMs with SNARF-1 are inside the amphipods, the final acquired spectrum in the range of 600–650 nm is formed by 3 stable fluorescence spectra: the spectrum of protonated SNARF-1, with a peak at 610 nm (in the optical system used); the autofluorescence of the amphipod (probably due to lipofuscin), with a peak at 627 nm; and the spectrum of deprotonated SNARF-1, with a peak at 640 nm (Fig. 2b). Because of this, the recorded *in vivo* spectra were found to be easily decomposable to these three parts by multiple regression analysis.

After decomposition of every acquired spectrum by multiple regression analysis, the background autofluorescence was subtracted to calculate the 605 nm/640 nm ratio from the SNARF-1 spectra and to apply the calibration curve to the *in vivo* pH measurements.

### *In vivo* monitoring of haemolymph pH

**Figure 3:**
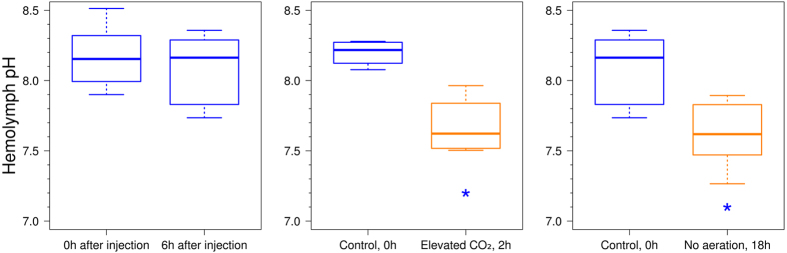
*In vivo* monitoring of the haemolymph pH of *E. verrucosus* under different conditions: right after the injection of MBMs and 6 h after the injection; under elevated CO_2_ level; and exposure without aeration. The blue colour indicates acclimation conditions, whereas orange indicates stress exposure. *Statistically significant differences from the control with a *p*-value < 0.05.

The measured pH values under the control conditions agreed well with the pH value of approximately 8.17, identified using a micro-pH-meter. To verify that the haemolymph pH value is not affected by the injection of MBMs itself, spectral measurements were taken right after the injection and after the incubation for 6 h in the acclimation conditions. The results show that, during the 6 h after the injection, there was no statistically significant (*p*-value = 0.674) change in the haemolymph pH and that the range of the median remained stable from 8.15 to 8.22 (Fig. 3a).

To determine whether the proposed technique will be able to detect physiological alterations of haemolymph pH, we carried out two types of exposures of endemic amphipods *E. verrucosus*, which can be used in the future for environmental risk assessment in the nearshore zone of Lake Baikal: elevated CO_2_ level in water, which should lead to hypercapnia; and exposure without aeration, which should lead to the combination of mild hypoxia and mild hypercapnia.

Results of the effect of elevated CO_2_ concentration on the pH of the haemolymph in amphipods, measured *in vivo* by the SNARF-1-containing MBMs, are shown in Fig. 3b. After 2 h of exposure to 55–70 mg of CO_2_/L, *E. verrucosus* showed a statistically significant (*p*-value = 0.029) decrease in haemolymph pH from the 8.1–8.3 range in control conditions to the 7.5–7.9 range after treatment by elevated CO_2_. This experiment shows the general suitability of this technique for *in vivo* pH measurements.

Exposure without aeration, which was considered a more environmentally realistic test than elevated CO_2_ concentration, also led to a statistically significant (*p*-value = 0.017) decrease in the median pH from 8.16 to 7.62 (Fig. 3c).

### Stress assessment with lactate content

**Figure 4:**
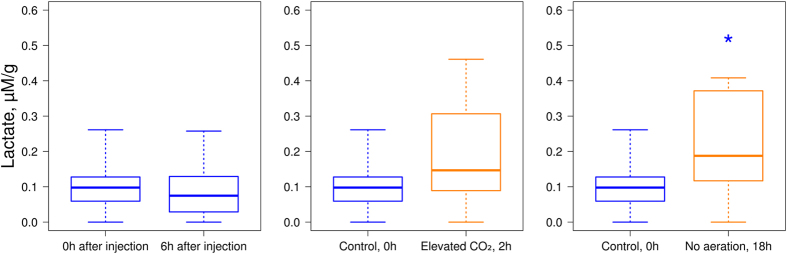
Dynamics of lactate concentration under different conditions: right after the injection of MBMs and 6 h after the injection; under elevated CO_2_ level; and exposure without aeration. The blue colour indicates the acclimation conditions, whereas orange indicates stress exposure. *Statistically significant differences with a *p*-value < 0.05.

In parallel, the stress reaction of endemic Baikal amphipods under elevated CO_2_ level and exposure without aeration was analysed using the lactate content, a common biochemical stress marker. However, before the stress exposures, the influence of the MBM injection itself on this biochemical marker was evaluated. Over the 6 h after the injections there were no significant changes (*p*-value = 0.915) in lactate content (Fig. 4), which allows us to remove from consideration the influence of the injections on amphipods during the further stress exposures.

Incubation in elevated CO_2_ level for 2 h did not lead to statistically significant change (*p*-value = 0.112) in the median lactate content (Fig. 4). Nevertheless, the variation in lactate concentration increased approximately 1.8-fold, which was not observed after injection and incubation in acclimation conditions.

Exposure without aeration for 18 h caused a statistically significant (*p*-value = 0.031) increase in the level of lactate.

## Discussion

Aquatic animals used as common test objects in environmental sciences are often small in size^31^. Current methods in this field usually do not allow for the identification of internal parameters of a small organism without the destruction of the animal. This forces researchers to use different individuals for physiological measurements at different exposure points, which automatically decreases precision due to the variability between individuals. In addition, the large number of animals required decreases the speed of analyses and increases the cost of studies.

In the current study, we overcame this problem by using MBMs for internal pH measurements in the same individuals over a range of time. Despite the high variability in the pH values measured by MBMs, in acclimation conditions, the median pH stayed in the 8.15–8.22 range over a series of experiments, which is in agreement with the average haemolymph pH 8.17 measured by micro-pH-meter in *E. verrucosus* and the pH values of up to 8.2 reported for some other crustaceans^20^,^32^.

Biochemical stress markers such as lactate (accumulation of which indicates a metabolic switch to anaerobiosis^33^,^34^) have previously been shown to react to a variety of stressors, such as different toxic substances, hypoxia or heat shock. Importantly, in the case of *E. verrucosus*, this and other markers generally immediately react within the first hours of stress exposure^26^,^35^,^36^. Here, we measured lactate content to both determine whether the injections of MBMs induce a stress reaction in amphipods and identify their stress reaction under elevated CO_2_ level (should induce hypercapnia) and exposure without aeration (should induce combined mild hypoxia and mild hypercapnia). During the experiment, no changes in the lactate content were observed after the injection of MBMs. Considering the sensitivity of this marker^33^,^34^,^37^, this indicates that the proposed technique of MBM injection does not cause an observable non-specific stress reaction in the amphipods, and this technique may be applied for stress assessment.

To date, changes in physiological parameters (for example, histamine^38^, glucose^39^, sodium^40^ or lithium^41^ levels) remotely measured using different types of immobilised fluorescent sensors in mice were only intentionally induced by researchers via injections of the respective substances. In other words, no observation of the shift in a physiological parameter has been made under indirect influences, such as stress. Living organism is a complex system, and precision of the immobilised sensors could potentially be insufficient to recognise natural physiological alterations, especially considering a relatively high inaccuracy of fluorescent molecular probes, the working substances inside these sensors. I.e., possibility to use the immobilised fluorescent sensors for animal stress assessment has to be verified directly. The results of our work are the first data showing the applicability of immobilised sensors (in this case, MBMs) for the identification of stress-induced physiological changes.

Hypercapnia is known to cause apparent pH changes in haemolymph of crustaceans^19^,^20^. Therefore, exposure to elevated water CO_2_ level was first applied to verify the sensitivity of the SNARF-1-containing MBMs to pH changes *in vivo*. Values obtained with the MBMs allowed identification of considerable haemolymph acidification in *E. verrucosus* under these experimental conditions. Simultaneously, an increase in variation of lactate content was observed, which may indicate a switch to anaerobiosis in a part of individuals under this exposure.

Incubation without aeration (which should lead to combined mild hypoxia and mild hypercapnia) can be considered a more environmentally relevant experimental design than acute hypercapnic exposure, and this incubation was applied to verify the sensitivity of the MBMs in conditions more natural for Baikal amphipods. The administered MBMs showed acidification of the haemolymph in these experimental conditions as well. Exposure without aeration also induced increase in the concentration of lactate. The shift of this marker is a significant indicator of stress and generally accompanies the activation of stress response mechanisms^33^,^34^,^37^.

The coincident reactions of MBMs and lactate content in amphipods in the both types of exposure show the potential of MBMs as a novel tool for remote *in vivo* stress assessments in the environmental monitoring of Lake Baikal and, likely, other water bodies. The proposed technique can already be used in experimental research for the effective evaluation of the possible effects of eutrophication and global climate change on the fauna of different water reservoirs.

In the future development of this technique, expansion of the number of simultaneously monitored physiological parameters may permit a comprehensive analysis of the current state of an organism to be performed *in vivo* and may bring the field of environmental monitoring to a new level of sensitivity.

## Methods

### Object of research

The amphipod species *E. verrucosus* (Gerstfeldt, 1858), endemic to Lake Baikal, was chosen as the object of research. No specific permissions were required for the sampling or other activities, as the investigated species is not endangered or protected. Amphipods were caught by kick-sampling on the shore of Lake Baikal (approximately 0.2–0.5 m depth) near the village of Listvyanka (51°52′05.5″N 104°49′47.1″E), on the south-west coast of the lake during April-June 2016 (the water temperature was in range of 2–8 °C). The amphipods were transferred to the laboratory in thermostatic boxes and acclimated at 6 °C (the average annual temperature of the littoral zone of Lake Baikal^25^,^26^) in tanks with approximately 2.5 L of Baikal water and strong aeration. The water in the tanks was exchanged every 2 days, and after every exchange, amphipods were fed *ad libitum* with a dried and ground mixture of algae and invertebrates from the sampling place. During the acclimation and all experimental procedures, amphipods were kept in low daylight conditions to prevent the photobleaching of SNARF-1. Only adult (25–35 mm) actively swimming animals without exoparasites and visible damage were used for further analyses.

### Preparation of pH-sensitive MBMs

The sensor SNARF-1 conjugated with dextran (SNARF-1-D; Thermo Fisher Scientific, USA, D3304) was used as the pH-sensitive fluorescent dye. Microencapsulated biomarkers (MBMs) filled with SNARF-1-D were prepared as previously described^28^,^42^. First, the method of layer-by-layer assembly of oppositely charged polyelectrolytes allowed to form the polymeric shell around porous CaCO_3_ cores containing SNARF-1-D. Following the dissolution of the CaCO_3_ cores, SNARF-1-D remained inside the polymeric multilayer shell, which is permeable to low molecular weight substances and impermeable to the large molecules of dextran bearing the dye (Fig. 1a).

SNARF-1-D was originally co-precipitated into CaCO_3_ cores by mixing 2 mL of a 1.2 mg/mL SNARF-1-D solution with 0.615 mL of a 1 M Na_2_CO_3_ solution and 0.615 mL of a 1 M CaCl_2_ solution under strong stirring for 5 s followed by washing 3 times in deinonised water. The obtained porous cores were then covered with 12 layers of oppositely charged polymers: positive poly(allylamine hydrochloride) (PAH; Aldrich #2832315) and negative poly(sodium 4-styrenesulfonate) (PSS; Aldrich #243051). Each layer was deposited for approximately 7 min. After each layer, the microparticles were washed 3 times in deionised water and treated with ultrasonication for 3 min. For better biocompatibility of the MBMs^28^, a poly(L-lysine)-graft-poly(ethylene glycol) co-polymer (PLL-g-PEG; SuSoS, SZ34-67) was deposited as the last layer.

After dissolving the CaCO_3_ cores in 0.1 M EDTA solution (pH 7.0), the final structure of the MBMs corresponded to the formula (PAH/PSS)_6_/PLL-g-PEG with SNARF-1-D inside (Fig. 1a).

### Injection of MBMs and the immobilisation of amphipods under a microscope

During injection, each amphipod was immobilised with a wet polyurethane sponge^43^ at the acclimation temperature (6 °C). MBMs were injected using insulin syringes (needle diameter: 0.3 mm; BD Medical, USA) into the central haemolymph vessel in the dorsal part of the pereon (Fig. 1d). Five microliters of phosphate-buffered saline (pH 8.0) with approximately 60,000 microcapsules (containing SNARF-1-D) per 1 μL were injected into each individual. The average size of the microcapsules was approximately 4 μm.

For the visualisation of MBMs inside amphipods, a custom-made chamber was used (Fig. 1e). Each individual amphipod was placed in the centre of a glass chamber filled with Baikal water, immobilised by the negative pressure inside the plastic tubing, installed in the middle of the chamber and connected to a peristaltic pump. The water pumped from the chamber was directed back, and on the way to the glass chamber, it was cooled in a thermostat to keep the temperature in the chamber in the range of 5–8 °C. Such a system allows immobilisation and analysis of only one amphipod at once. An amphipod can be safely maintained in this chamber during for least several days.

### Experimental procedures

After acclimation, amphipods were subjected to 3 types of experiments, and for each type, the physiological parameters related to stress were monitored with either pH-sensitive MBMs *in vivo* or classical biochemical methods.

#### Effect of injection of MBMs on haemolymph pH

Acclimated amphipods were injected with MBMs, and the haemolymph pH was measured under the fluorescent microscope immediately after the injection. Following this, the amphipods were placed back in the acclimation conditions (6 °C with constant aeration), with each specimen separate from the others in 0.5-L glass tanks and incubated for 6 hours. After the incubation amphipods were placed under the microscope, and the signal was measured again. In parallel, a control group of amphipods was frozen in liquid nitrogen after acclimation for biochemical measurements, and another group of animals, injected with MBMs, was frozen in liquid nitrogen after 6 h in acclimation conditions.

#### Exposure to elevated CO_2_ level in water

For *in vivo* pH measurements, acclimated amphipods were injected with MBMs and immediately placed under the microscope for fluorescent signal recording. After this, animals were transferred for 2 h to a thermostatic chamber (6 °C) with Baikal water already saturated with CO_2_, and the signal was recorded again. The CO_2_ level was monitored during the exposure and fluctuated in range of 55–70 mg/L. These conditions were considered to be highly hypercapnic, as the measured level of CO_2_ in Baikal water is 1–2 mg/L. The oxygen level during the whole exposure was in range of 11–12 mg/L. For estimation of lactate content, two groups of amphipods (not subjected to injections) were fixed in liquid nitrogen immediately after incubation in acclimation conditions (control) and after 2 h of the subsequent hypercapnic exposure.

#### Exposure without aeration

After their haemolymph pH was measured *in vivo*, acclimated animals were kept in separate tanks with 0.5 L of Baikal water without aeration. At the end of the exposure, the O_2_ level at the bottom layer of the tanks had dropped from 11–12 to 8–10.5 mg/L (which may be considered as mild hypoxic conditions for Baikal endemics as the concentration of dissolved O_2_ in Lake Baikal is relatively high and can reach 14.5 mg/L^44^), and the CO_2_ level raised from 1–2 to 4–6 mg/L. After 18 h of such exposure, the haemolymph pH was measured again. For biochemical analyses, amphipods were deep-frozen after acclimation (control group) or after 18 h of exposure to combined mild hypoxia and hypercapnia.

For all experiments, adult amphipods with body sizes in the range of 25–35 mm were used. No mortality was observed during any of the experiments, and amphipods appeared to be actively swimming. The CO_2_ level was measured by a commercial test (Tetra, Germany) using NaOH titration in the presence of phenolphthalein according to the recommendations of the manufacturer, and the O_2_ concentration was measured with a dissolved oxygen metre (Model 407510, Extech Instruments, USA). Experimental groups for biochemical measurements included 9–13 individuals, for *in vivo* pH measurements 4–8 animals in each group were used. Statistically significant differences between experimental groups were verified by the Mann-Whitney test in package R^45^.

### Visualisation and calibration of pH-sensitive MBMs

Visualisation and signal acquisition from MBMs was carried out with the Mikmed-2 microscope (LOMO, Russia) in combination with the QE Pro spectrometer (Ocean Optics, USA; INTSMA-200 optical slit; integration time set to 1 s) through an F280SMA-A collimator (Thorlabs, USA) and QP400-2-VIS-NIR optical fibre (Ocean Optics, USA). The collimator was mounted in focus of the ocular lens and transferred the light from the whole field of view through the fibre to the spectrometer. Fluorescence measurements were taken using an excitation wavelength of 545 nm, and images and spectral signal were acquired in the red channel. The MBMs inside the amphipods were visualised with the 10× objective lens (working distance approximately 5 mm).

Before the injections, MBMs were suspended in a number of pH buffers within the pH range of 6.5–8.9 as well as at pH 2 for calibration. MBMs in the buffers were visualised with a 40× objective lens (the residual spectrum from the illumination source was minimal for this objective) and the spectral signal was recorded immediately after the sample was placed under light to minimise photobleaching. The ratio of the SNARF-1-D fluorescence intensity between wavelengths 605 nm and 640 nm was used to build the calibration curve and to measure pH *in vivo*.

### Analysis of the spectral signal from MBMs inside amphipods

Autofluorescence of the dorsal part of pereon of the investigated amphipods was found in the functional range of SNARF-1-D. To solve this issue, a multiple linear regression analysis was used to decompose the acquired spectra to 3 known components: the fluorescence spectrum of the protonated SNARF-1-D at pH 2, the fluorescence spectrum of the deprotonated SNARF-1-D at pH 8.9, and the typical autofluorescence spectrum of the dorsal part of the pereon of *E. verrucosus*. With the optic system used, these spectra have peaks at approximately 610, 627 and 640 nm, respectively, and the contribution of each spectrum to the measured spectrum is distinguishable. The multiple linear regression analysis was carried out in the spectral range of 600–650 nm using the Scilab package^46^ with the *regres* function from the Stixbox toolbox. Constants were not allowed in the regression model.

After building the regression model, the autofluorescence spectrum was subtracted from the original spectrum to calculate the intensity ratio for SNARF-1-D as was performed for the calibration curve. This ratio was indicative of the haemolymph pH inside the amphipod circulatory system.

### Analysis of lactate content and measurements of haemolymph pH

The concentration of lactate was determined in the whole body of *E. verrucosus* using the express kit “Lactate-vital” (Vital-Diagnostics, St. Petersburg, Russia) according to Bergmeyer^47^. Measurements were performed with the “Cary 50” spectrophotometer (Varian, USA) at λ = 505 nm.

Haemolymph pH of *E. verrucosus* at acclimation conditions was measured by a microelectrode (InLab Nano, Mettler Toledo) at approximately 6 °C right after haemolymph extraction.

## Additional Information

**How to cite this article**: Gurkov, A. *et al*. Remote *in vivo* stress assessment of aquatic animals with microencapsulated biomarkers for environmental monitoring. *Sci. Rep*. **6**, 36427; doi: 10.1038/srep36427 (2016).

**Publisher’s note:** Springer Nature remains neutral with regard to jurisdictional claims in published maps and institutional affiliations.
